# Dr. Huggan, on Venæsection, &c. in Reply

**Published:** 1800-06

**Authors:** A. Huggan

**Affiliations:** Plymouth


### *To the Editors of the Medical and Physical Journal.*

GENTLEMEN,

I Take the liberty of sending you this letter, in answer to three of your correspondents, who have animadverted on my opinions, respecting the use of opium and venæsection, in the practice of medicine, which I will thank you to insert, if you think it worthy of a place, in a future Number of your Journal.

I pass over, without any notice, what Mr. Urvins, the first of these gentlemen, has in the preface to his remarks, hinted by way of caution to your readers, against the reception of *hasty* or *extravagant notions*, which I suppose mine are thence implied to be, as also his oblique imputation of enthusiasm to the encomiums which I have bestowed upon opium in the cure of febrile diseases.

If this gentleman thinks he can see any thing like argument to the purpose, from the conceit of any person's being equally justified in recommending the complete expulsion of opium from the *list of remedies*, because he may have, by accident, been a witness to its fatal effects, or the mis-application of it, it is sufficient to answer, that I have not contended against venæsection from the consideration of its ill effects *only*, but that under any circumstances, even the most favourable to the employment of it, as a means of cure, I do venture to affirm that it is never necessary. This, in whatever light it may appear to Mr. Urvins, is no hasty or extravagant notion, but what I have ascertained to my complete satisfaction, at least as far as I can trust to the facts and observations on which this conclusion is grounded. Venæsection may therefore, in my opinion, be as safely and as advantageously superceded in inflammatory fevers, as in any other disease, in which its disuse has been acknowledged as an improvement.

With regard to venæsection in croup, I have, in a former part of this Journal, (Numb. XI. p. 56.) briefly stated my reasons why I do not think it even admissible; these were the result of some experience, much reflection on the danger of bleeding children, at the early age at which this disease generally makes its appearance; as also the infirm state of health of several of my acquaintances, whom in early life I can recollect to have been subject to croup, and, on that account, occasionally blooded. To these I shall here add, that, if it is contagious, as has been alleged, this would weigh with me as a further objection against the employment of the lancet.

As

As Mr. Purton has had much experience in the treatment of croup, I dare ſay that he has ſometimes obſerved the ſymptoms, after they had been alleviated by means of the lancet, return with redoubled violence, and carry off the patient before any aſſiſtance could be afforded him.

The relief obtained in croup from opium, has certainly appeared to me to have been more permanent, and the recurrence of the diſeaſe leſs violent, than after the employment of venæſection. If opium is to be truſted as our principal remedy in croup, I muſt again repeat it, the doſe of it muſt be proportionate *to the violence of the diſeaſe*; and, what is of equal importance, muſt be adminiſtered early in the diſeaſe, otherwiſe it will be equally as inefficacious, in averting the impending danger, as any other means which we can deviſe; for, as Dr. Ferriar juſtly obſerves,* "If the alarming ſymptoms are not mitigated during the firſt ſix hours, the diſeaſe generally proves fatal." As, we learn from the ſame reſpectable authority, that "in the caſe of very young children we muſt almoſt deſpair, for it is extremely difficult to procure any blood from them by the lancet, and leeches afford a very inadequate mode of depletion." Under ſuch circumſtances then, and eſpecially after the ſecond bleeding and emetic have failed in putting an end to the diſeaſe, when we are told that "we have nothing to hope from medicine," ſurely the humane phyſician may, without incurring any riſque of cenſure, have recourſe to opium, rather than leave his patient unavoidably to periſh in the unequal ſtruggle with ſo formidable a diſeaſe as croup; and if he ventures to adminiſter it with freedom, for once, perhaps, he may not be diſappointed. Not only does this learned author ſtrenuouſly injoin venæſection in croup, but alſo ſanctions a licence in performing it, to me altogether

* Medical Hiſtories and Reflections, by J. Ferriar, M. D. Vol. III. Cadell and Davis, in which the reader will find a very accurate deſcription of Croup, ſuch, indeed, as might be expected from the pen of that learned writer:

About three years ago I was deſired, a little paſt midnight, to viſit a boy, about nine years old, whom I found expiring in a fit of the croup; I was told, that after a ſlight indiſpoſition of two or three days, he had been attacked the preceding evening in a ſimilar manner, for which he had been directed to take an emetic, and ſeemed to be better in the courſe of the day, till towards evening he was again ſeized as I found him. On diſſection, I found ſome of the branches of the trachea completely filled with a membrane-like exudation, which had entirely cut off all communication with the external air; the aorta, and its larger branches, were lined with a membrane of the ſame appearance. Had proper remedies been applied on the firſt ſevere attack, this boy would have had a better chance for his life.

together unjustifiable; the surgeon is told not to be scrupulous about the appearance of *mangling* in circumstances so dreadful. Now, if the arm is made choice of for that purpose, we know from anatomy, with what caution this nice operation must be done, to avoid doing mischief. Would it not be better in such cases, in which blood-letting is held to be indispensibly necessary, to open one of the small branches of the external carotid artery, than run the risque of doing an irreparable injury, by rudely performing an operation, that requires the greatest delicacy and steadiness on the part of the surgeon, and which, at best, is seldom or never performed in the nice manner that it ought to be.* Were I again to have recourse to venæsection, I would generally prefer the external jugular vein; as here, the orifice may be made with less risque to the patient; it closes again as readily, and the blood will flow in a larger stream than from the veins of the arm or leg, therefore a less quantity will suffice.

Mr. Purton having insinuated rather too hastily, that the cases of croup, which fell under my observation, must have been of the spasmodic kind, if they yielded to opium before a general depletion had been made; I shall just observe in answer, that though the inflammatory and spasmodic, may, in their less distinct forms, often with difficulty be distinguished from each other, yet, there are sufficiently unequivocal marks by which a case of genuine cynanche trachealis may, I think, always be known. The cases to which I alluded chiefly, were first attacks, which, I believe, are always inflammatory. Indeed, I think it probable, that the spasmodic croup never occurs as a primary disease, and, perhaps, is to be met with in those instances only, in which the inflammation had extended from the inside of the trachea to the neighbouring muscles; it ought therefore, to be regarded as a variety, or the consequence, than as a distinct species of croup.

I shall conclude this part of my letter, with expressing my regret, that the venerable father of physic, when he adverted to the inutility of *antispasmodics*, in preventing the fatal issue of this disease, had not sufficiently proved the efficacy of the most powerful of that class of medicines. (First Lines of the Practice of Physic, CCCXXX) I come next to Mr. Dray's criticisms, which he too, like Mr. Urvins, has thought proper to introduce with an admonitory hint, nearly of the same tendency.

This gentlemen, in his first animadversion, points out, what he

* B. Bell's Surgery, Vol. I. Ch. on Blood-letting.

he conceives to be an inconſiſtency, or contradiction of myſelf, which he infers from the theory that he has been pleaſed to impute to me, as "the *baſis of all my practice*;" and the aſſurance which I had given, in the outſet of my communication, that wherein my practice might differ from that of others, it was entirely the reſult of experience, as I had no *theory to ſerve*; by which was ſimply meant, that, what I was about to ſubmit to the conſideration of the public, was founded on the ſure baſis of experience, as I had no fanciful or deluſive ſpeculations to gratify. The *debility* which accompanies every deviation from the healthy ſtate, and is the unavoidable conſequence of increaſed action of the ſyſtem, was held out ſolely with the view of precluding venæſection, or whatever tends to debilitate further, as a matter of courſe; but this, ſurely, was not inſiſted upon, as the principal, or only circumſtance, by which our method of cure ought to be regulated; for I am equally aware with that gentleman, how *truly incongruous* a practice, built upon ſuch vague and unſatisfactory grounds, muſt be. I ſhall here, however, briefly ſtate, for it can be done in a few words, what really is my theory of inflammatory fevers, and leave it to the enlightened and unprejudiced part of the profeſſion, to decide how *properly* it is adapted to the practice which I have propoſed. In inflammatory, or fevers with arterial ſtrength, there is, it is preſumed, an exceſs both of ſtimulus and ſenſorial power.* Now opium, exhibited in ſuch a doſe as to afford a greater ſtimulus than that of the inflammatory pains, will ſpeedily and effectually exhauſt the ſenſorial power, or the greateſt part of it; hence, if the ſpaſm of the extreme arteries is not overcome, it will ceaſe to excite the parts affected, or the whole vaſcular ſyſtem into inordinate action, from defect of ſenſorial power.

By venæſection, if copious, the activity of the whole ſyſtem, from the *ſudden* loſs of part of that important fluid which conveys nutriment and ſtimulus to all the parts of it, is inſtantly diminiſhed; whereby ſome of the functions, particularly that of the brain, are as ſuddenly impaired, or ceaſe entirely, as appears from the drowſineſs or fainting that often follows great and ſudden loſs of blood; hence the protracted period of diſeaſes, and ſlow recoveries, after the free uſe of the lancet. So far from theory alſo, we think the preference due to opium, as leaving the ſyſtem in a leſs debilitated ſtate, after its action, than venæſection.† It

* Zoonomia, Vol. I. Sect. Stimulus and Exertion; Vol. II. Theory of Fever, Art. Incitantia.

† Such of your readers as are not quite diſguſted with the French bombaſtic

It is unneceſſary to follow Mr. Dray through all his reaſoning, as, from the *data* only which he has aſſumed, that muſt be inconcluſive. I ſhall not either contend with him about the propriety of changing the mode of treatment, in the caſe of the military men which he mentioned, that had been adopted previous to his attendance; as it muſt be plain to every diſcerning practitioner, that, under the circumſtances as he ſtates them, a plan of cure, conducted with a view to the concomitant debility *only*, muſt have been obviouſly abſurd, and the iſſue, as might be expected, generally fatal. I find, however, that we agree ſo far in our ideas of the treatment neceſſary in inflammatory fevers, as, that we both think it right, to employ "means which diminiſh increaſed excitement;" our only difference of opinion ſeems to be, whether theſe *means* ought to be ſuch as ſuddenly increaſe the ſtimulation, ſo as to exhauſt the ſenſorial power completely, or ſuch as ſuddenly diminiſh both. There is alſo a difference of opinion between us reſpecting the effect of bliſters, which he is inclined to think are apt to increaſe the ſymptoms, when applied before "the inflammatory diatheſis is ſufficiently ſubdued." I conſider the action of bliſters to be merely local, and, as I barely hinted in my Inaugural Diſſertation on the Influenza, they produce their effects ſolely, by exciting a greater irritation in the part than that for which they are applied. I can therefore ſee no impropriety, nor did I ever experience the ſmalleſt increaſe of the ſymptoms, from the early application of them in pneumonia, or other caſe of phlegmaſia.

Mr. Dray's arguments, taken from the practice of the ancient phyſicians, as well as his own experience, and that of the ableſt practitioners of our own day, allowing them their full force, go only to prove that venæſection has been, and may ſtill be, employed in phlegmaſia with advantage, ſo far as to remove the danger with which they are attended. This has never, to my knowledge, been denied; but they do not invalidate the teſtimony which I have given, or overthrow the doctrine that it holds out in favour of a method of cure, in theſe diſeaſes, in which venæſection may, with ſafety, and evident advantage, be omitted.

All that has been urged againſt the early exhibition of opium in ſynocha, &c. have, in my opinion, ariſen almoſt ſolely from prejudice, or the miſapplication of it in ſome ſhape or other;

---

baſtic accounts of their battles, their robberies, and their travels, may find, in the 3d Vol. of Sonnini's Travels in Upper and Lower Egypt, p. 68, a remarkable inſtance of a ſevere ſpecies of ophthalmia being cured by an over doſe of opium, after a copious bleeding had failed in giving any relief.

other; hence effects have been imputed to it which it does not produce, and explanations given of its action, equally erroneous.

This remark is occasioned, principally by what has been written on this subject by the illustrious author of Zoonomia, (Vol. II. Article Incitantia, p. 2, 1, 6.) where we are told, that, in the cases of phlegmasia, in which opium gives relief, it acts first by increasing the pain, and is sometimes followed by so great a torpor, as to produce "the death or mortification of the parts;" hence we are cautioned against its use, in inflammations, particularly that of the bowels; and further, that the relief thus obtained, is not permanent, as the pain is said to return with its former violence.

With the utmost deference, however, to so great authority, I must here take the liberty of saying, that I suspect these to have been assumed, as the effects of opium, in conformity to the learned author's general doctrines, or perhaps, from instances of timid or injudicious exhibition of it, rather than clearly demonstrated by any fair or decisive trials of it. Thus far at least, I have presumed, from my own experience. I have not met with an instance of relief, in inflammation being procured by opium in a full dose, in which the pain was first increased, though this has been looked for. When given in small doses indeed, as I have observed, it will not fail to increase the pain, by adding to the stimulation already too great, as well as producing a still greater quantity of the spirit of animation; by which means, the vital powers of the system will be worn out by such inordinate action. In one case of enteritis, which I have met with, six grains of opium, given in two doses, produced the happiest effects. I have certainly observed the return both of inflammatory and colic pains after relief had been obtained by opium, but in no one instance with the same violence.

I have been the more readily induced to meet this celebrated physician's objections to the exhibition of opium in the first stages of phlegmasia, (they would have been unanswerable if well founded) as, upon the same grounds I can, with confidence, correct another statement of his, respecting opium, which he has certainly assumed merely from conjecture.—In the beginning of the same paragraph, he says, that this relief does not occur till some hours after the exhibition of opium. I know from frequent observation, that opium, if given in a sufficient dose, will produce its utmost effects in less than an hour; when it fails in that time, we may be assured that the dose has been too small, and ought to be repeated in the same, or half the quantity previously administered. In slighter cases of inflammation indeed, (in ſuch only, I ſuſpect, has opium ever been ventured upon, in the firſt inſtance) in which relief has been procured ſome hours, after the exhibition of opium, it ſeems to have acted, by aſſiſting the other ſtimuli, in reducing the ſenſorial power to the natural quantity, rather than by exhauſting it entirely by its own ſpecific powers.

I ſhall not, at preſent, enter into any further diſcuſſion of the propriety of ſubſtituting opium in the cure of ſynocha, &c. inſtead of venæſection, as, in the end, that muſt be decided by experience *alone*; to that teſt then, in the hands of the liberal and intelligent part of the profeſſion, as far as I am intereſted in the iſſue, I do moſt cheerfully appeal. Whether, however, the practice which I have ſo ſtrenuouſly recommended, be ever adopted or not, I feel conſcious that it was not communicated to the public, with any view of entering into a controverſy on the ſubject, or from any hope of being able to build my fame on the reception it might meet with, as that is not at all neceſſary; nor from the affectation of ſingularity; but ſolely, from the perſuaſion and experience of its being the preferable and ſafeſt plan; and with the ſincere wiſh, that it may prove, in the hands of thoſe who may be inclined to give it the trial, as ſatisfactory as, in the courſe of nearly five years, it has been in mine.*

I ſhall

* As Mr. Dray ſeems to think my " opinion in its nature ſo calculated to miſlead young and inexperienced practitioners, as to be productive of the moſt fatal conſequence," I may, with equal juſtice, reply, that it cannot be of leſs dangerous tendency to allow ſo unlimited a power in the uſe of the lancet, as to authoriſe, not only ſtout athletic adults, but even infants, to be blooded ad deliquium. The lancet therefore may become a formidable weapon, when wielded by ignorance, inexperience, or enthuſiaſm. When I was about eleven years old I was thrown from horſeback, and pitched upon my head; and, had I been permitted to lay where I fell for a few hours, till I had recovered from the ſtate of inſenſibility in which I was carried home, I might probably have eſcaped the ſevere diſcipline which I afterwards underwent. A bleeder was ſent for immediately, who, in addition to what I had loſt, from the coronary artery of the lips, which was cut through, took away a ſoup plate full of blood from each arm; having very careleſsly bound up the orifices, they were burſt open, a few hours afterwards by the agitation of vomiting, and continued to bleed again till I almoſt fainted. The effects of ſo great a loſs of blood I felt for years afterwards.

When I was very *young* and *inexperienced* in the profeſſion, having juſt laid out of my hands Sharp's Critical Inquiry into Surgery, in which I had been reading, that in no complaint (pleuriſy excepted) was bleeding more uſeful than in hernia; I happened to be ſent for to a ſtout man, about fifty years of age, who had been ſeized with this dreadful diſorder, not however in a more violent degree than he had ſometimes experienced it before. Towards morning all the ſymptoms of ſtrangulation were coming on rapidly. Encouraged by ſuch authority as that of Mr. Sharp, I immediately took away about

I ſhall not take up your time, with endeavouring to refute Mr. Dray's oblique inſinuation of preſumption, implied in the firſt part of p. 328; as it does not appear, from any part of my communication, to have been merited; nor ſhall I cavil about the degree of weight *only*, which he allows to the caſes which I ſelected, from ſeveral others of a ſimilar nature. I ſhall only explain more particularly, ſome circumſtances, to which he has very erroneouſly aſcribed a greater ſhare of the cure in the caſes of contuſion than they merited. In neither of them was the loſs of blood from the contuſed parts ſo great as either to promote or retard the cure in the ſmalleſt degree. He is equally miſtaken, in the effects, which he ſuppoſed the calomel to have been given, in the caſe of Pneumonia, or in any of the others, with the view of producing. As mercury, in whatever manner its mode of action is to be explained, has been found to aid the efficacy of other remedies, it is upon the ſuppoſition of its increaſing, or promoting, the anodyne powers of opium, that I generally preſcribe calomel along with it. In injuries from external violence, eſpecially of the head, I have been in the habit of directing the patient to have a briſk cathartic, after the effect of the opiate has ceaſed, not from any view to evacuation as being at all neceſſary, but for the purpoſe of exciting a different action in the ſyſtem, if that can, in any degree, avert the danger that may be dreaded; this, I allow, may be more imaginary than real; it is only a ſecondary conſideration.

I ſhall

---

about twenty ounces of blood from a large orifice, by which he was certainly much relieved; but on the following evening he died, with every appearance of the fatal event having been haſtened, if not occaſioned *ſolely*, by the loſs of ſo much blood.

I have lately been induced to inquire more particularly into the effects of "forty to fifty and ſixty ounces of blood" being taken away "in a couple of hours, (Medical and Phyſical Journal, Vol. II. p. 284.) and that in dyſentery too, in which I have always underſtood that the lancet ought to be uſed with caution. Nay, as the author, as well as his aſſiſtants and his patients have told me, upwards of a hundred ounces have been taken in the courſe of a few hours. How many "valuable lives" were not loſt by ſuch treatment, can only be inferred from other complaints having been miſtaken for, or fancied to have been, dyſentery. The reſult of my inquiry, however, was neither gratifying to my own feelings, nor at all encouraging to adopt this author's practice.

This evacuating plan, incredible as it may appear, is even trifling to that of the Tranſatlantic practice of phyſic, (Ruſh's Medical Inquiries and Obſervations, &c. Vol. IV.) No matter what the theory is, the author generally contrives to make it correſpond to his practice. If the *arteries* are *convulſed*, blood muſt be drawn to check their inordinate action; or, if the blood veſſels are morbidly diſtended with "ſeptous gas," an opening muſt be made to let it eſcape. The conſequences of ſuch practice muſt appear *obvious* to every enlightened phyſician, at leaſt on this ſide of the Atlantic.

I ſhall not take notice of what Mr. Dray has advanced, reſpecting the treatment of certain caſes of Ague, with any intention of criticiſing either his opinions on the ſubject, or his practice; but to recommend to him another remedy, perhaps leſs objectionable than the cinchona, and, I hope, I am not too ſanguine in adding, equally as efficacious; I mean, the broad-leafed willow bark. It will alſo give me an opportunity of acknowledging, to Mr. White, of Bath, how much I feel myſelf indebted to him, not only for his publication on the ſubject, but alſo for the benefit which I have myſelf experienced from its uſe.

I have for ſeveral years been ſubject to attacks of a quartan, and which I hardly dread ſo much as the diſtreſſing effects of the Peruvian bark, which it never fails to produce, if I take it in ſubſtance, even in very ſmall doſes. About two months ago I had a ſevere attack of ague, accompanied with vertigo, &c. the paroxyſms of which were removed by an ounce of willow bark in powder, taken in the courſe of forty-eight hours; I was alſo agreeably ſurpriſed with finding, during this period, my appetite much increaſed; indeed, I did not experience a ſingle unpleaſant effect, except coſtiveneſs, from it. This ſuggeſted to me a hint, of the probability of its further utility; I have therefore tried its efficacy in the incipient ſtates of convaleſcence, to promote the appetite, in a doſe of half a drachm, an hour before breakfaſt, and the ſame before dinner, and with as much ſucceſs as could reaſonably be expected. I have alſo experienced the ſame good effects from its uſe, in ſeveral caſes of Diarrhœa; one, of a child ſeventeen months old, who was very much emaciated from the ſeverity and duration of the complaint. The quantity taken was two drachms of the powder in the ſpace of three days. Though I agree with Mr. Dray, that "a few caſes only, ought not to ſatiſfy any practitioner," yet I think they are ſufficient encouragement for myſelf to make further trials of it, and to recommend the ſame to others of the profeſſion. My own is the only fair caſe of ague, in which I have had an opportunity of adminiſtering it ſince I met with Mr. White's publication. It is indeed much to be wiſhed, that its efficacy in curing agues could be fully eſtabliſhed, for other reaſons than merely that of its making a very valuable addition to our liſt of febrifuge remedies, or of a great ſaving being thereby made in the neceſſary expenditure of public charities. That claſs of people who are moſt expoſed to the cauſes of ague, are, in general, the leaſt able to procure the means neceſſary to obviate either the diſeaſe itſelf or its too baneful effects, many of whom, from ſuch a cauſe, are yearly loſt, if not irrecoverably, at leaſt for a conſiderable time, to to their friends and the public. It would therefore tend, in ſome, I hope in no ſmall degree in numerous inſtances, to alleviate, if not to prevent entirely the accumulation of human miſery, from diſeaſe as well as poverty, if a remedy could be found growing by the ſides of our own rivulets, of equal efficacy with that which we are obliged to purchaſe at a great price, and from a diſtant clime, in reſcuing no ſmall portion of our fellow-creatures from the clutches of "a meagre fiend," which,

"With feveriſh blaſts, ſubdues the ſickening land."

As I have often witneſſed much diſtreſs from long continued ague, and the poverty of the patients who were afflicted with it, I am the more earneſt, on that account, in recommending trials of the broad leafed willow bark, as a ſubſtitute for the Peruvian, by others of the profeſſion, to whom favourable opportunities for that purpoſe may occur; notwithſtanding it has been ſo flatly aſſerted, perhaps on very ſlight grounds, that, "in point of efficacy, it is in no degree to be compared with the Peruvian bark." (Edinburgh New Diſpenſatory, 1797, p. 233.)

I ſhall conclude this letter with a little ſpeculation on the oxygenation of the blood, which, if not well founded, will, I hope, do no harm. This proceſs is generally ſuppoſed to be carried on by the oxygen of the atmoſphere paſſing through the moiſt membrane of the lungs, without any *peculiar* action on their part, but entirely from its being attracted by the blood. This theory is neither ſupported by analogy, for here, demonſtration is altogether out of the queſtion; nor does it, in my opinion, explain why, under the ſame atmoſphere, the blood of one perſon becomes ſuperoxygenated, as in ſynocha, conſumption, &c. or ſuboxygenated, as in hæmorrhea petechialis, confluent ſmall-pox, &c. or why ſimilar changes take place in the ſtate of the blood in the courſe of a few hours, in the ſame perſon, as I ſuppoſe to happen in the cold and hot fit of fevers. It may therefore, I think, be fairly preſumed, that the oxygenation of the blood, inſtead of being a mere chemical proceſs, is carried on by an appropriate ſet of organs, which, from their minuteneſs, and the office which they have to perform, will ever elude the niceſt inveſtigation of the anatomiſt; and, that it ought to be regarded as one of the animal functions,—that, indeed, on which all the others depend. If this is granted, I can carry my ſpeculations to the production of diſeaſes accompanied with theſe different degrees of oxygenation, as alſo, to the action of ſome of the remedies commonly employed. We ſuppoſe then, that the cold fit of fever is occaſioned by a torpor of theſe organs, which, from their office, may may be called INHALENTS, whereby the blood is not supplied with its *usual* or *necessary* quantity of oxygen; hence, the death of persons who have died in this stage of fevers may be accounted for, not merely from the heart becoming torpid, by association with other parts of the body, but chiefly from a defect of this stimulus in the blood. Upon the same principle, I suppose, that some noxious powers induce disease, by affecting the *inhalents* either primarily, by being *directly* applied to them, as in contagious diseases, pneumonia, &c. or *indirectly* by their association with other parts of the system, as in the small-pox from inoculation, scurvy, fever from external violence, &c. Conformably to this opinion, it is supposed, that those medicines which affect the oxygenation of the blood, act, not by forming a chemical union with either of its constituent parts, or by communicating or depriving it of oxygen, but solely, by increasing or diminishing the powers of the inhalents, whether they are applied *directly* through the medium of the atmosphere, as suggested and practised by the justly celebrated and ingenious Dr. Beddoes, or act by association, as exemplified by digitalis, opium, &c. exhibited by the mouth, mercurial frictions, &c. If this doctrine shall hereafter be found to be just, it will become of importance to study, with what parts of the system the action of these vessels is most particularly associated, that we may administer our remedies with better effect, and guard more effectually against the exciting causes of disease. Though this theory has long been the favourite amusement of my leisure hours; yet, I durst hardly have indulged my speculation so far, as to hazard public criticism, had I not met with somewhat of a similar opinion in the great M. Fourcroy's Memoir on Pneumatic Medicine, in the 2d Number of your very useful Miscellany.

I am, GENTLEMEN,

Your humble servant,

*Plymouth, May* 1, 1800. A. HUGGAN.

P. S. Since the foregoing letter was written, I have had an opportunity of perusing the 15th Number of the Medical and Physical Journal; wherein I find a Cynical animadversion on my opinions, in Mr. Christie's communication, p. 455, which I wish to notice, not for any *argument* that it contains, but only to say, that by his sarcastical allusion of *originality*, he gives me more credit, in that respect, than I deserve. I was induced by authority sufficiently respectable, and reasoning that appeared to me, as experience has since proved it, equally conclusive, to chuse the lancet entirely. I feel neither so intoxicated with the subject, as to have placed *my own conceits* "in a conspicious situation," as real matter of fact, or to have given the the hasty result of a *few* indecisive trials, as that of steady and attentive experience and observation. Nor need I be deterred from it, by the fullest analysis and comparison of "the materials with the solid structure of truth, of old industry, of extensive experience, of candid reasoning, of ripened talents." This I should rather wish than fear to meet, as I am confident, that neither *cruel disappointment* nor *fatal deception* will so often follow the practice therein suggested, if adopted to the extent which is recommended, as that for which Mr. Christie is equally as strenuous an advocate.

Mr. Dunning's query (p. 439) may be answered in the negative, as one of my cow-pox patients has, about a fortnight ago, had the measles.

